# Phenotypic and genotypic characterization of clinical *Staphylococcus lugdunensis* isolates: a French retrospective cohort study

**DOI:** 10.1128/spectrum.03856-25

**Published:** 2026-06-24

**Authors:** Marin Delaunay, François Gravey, Olivier Join-Lambert, Yohann Repessé, Sandrine Dahyot, Martine Pestel-Caron, Jean-Christophe Giard, Renaud Verdon, Pascal Thibon, Simon Le Hello, Christophe Isnard

**Affiliations:** 1 Université de Caen Normandie, Univ Rouen Normandie, Normandie Univ, INSERM, DYNAMICURE UMR 1311 27003, Caen, France; 2 Department of Infectious Agents, Bacteriology Unit, Université de Caen Normandie, Univ Rouen Normandie, Normandie Univ, INSERM, DYNAMICURE UMR 1311, CHU Caen Normandie 26962, Caen, France; 3 Department of Biological Hematology, Université de Caen Normandie, INSERM, PhIND UMR1237, GIP Cyceron, CHU Caen Normandie 26962, Caen, France; 4 Université de Caen Normandie, CHU Caen Normandie, National Reference Center for Willebrand Disease, Caen, France; 5 Department of Bacteriology, Univ Rouen Normandie, Université de Caen Normandie, Normandie Univ, INSERM, DYNAMICURE UMR 1311, CHU Rouen 55052, Rouen, France; 6 Department of Tropical Medicine and Infectious Diseases, Université de Caen Normandie, Univ Rouen Normandie, Normandie Univ, INSERM, DYNAMICURE UMR 1311, CHU Caen Normandie 27003, Caen, France; 7 CHU Caen Normandie, Centre d'appui Pour La Prévention Des Infections Associées Aux Soins, CPias Normandie 26962, Caen, France; Icahn School of Medicine at Mount Sinai, New York, New York, USA

**Keywords:** *Staphylococcus lugdunensis*, infective endocarditis, von Willebrand factor, lugdunin genomic study, virulence factors, antibiotic resistance, prosthetic joint infections, skin and soft tissue infections

## Abstract

Despite its dominance as a commensal, *Staphylococcus lugdunensis* (SLU) is a particularly virulent coagulase-negative *Staphylococcus* that is predominantly associated with community-acquired soft-tissue and skin infections. The aim of our study was to identify genotypic and phenotypic traits associated with the clinical presentations of SLU-associated infections, such as prosthetic joint infections, infective endocarditis (IE), and skin and soft tissue infections. Between January 2001 and December 2016, 73 pathogenic (including 18 strains responsible for IE) and 3 nonpathogenic SLU strains were retrospectively collected from nine French hospitals. All the strains belonged to six clonal complexes (CC1 to CC6) and 12 sequence types (STs). Interestingly, the capacity of the IE-associated strains to bind the von Willebrand factor was greater than that of the other strains, regardless of the CC. Biofilm formation was significantly increased for the most common CCs involved in infections (CC1, CC2, and CC3) and for IE-associated strains. The ability of SLU to inhibit other pathogens varied depending on the CCs caused by specific mutations within the *lug* operon that resulted in truncated protein expression. Genetic variants from genome virulence genes such as *agrC* and *atIL* also vary according to STs. The *Galleria mellonella* infection model revealed that CC4 was more virulent than the other CCs. On the basis of a large sequenced collection of SLU strains, we found that important phenotypic variation occurred among clinical strains and seemed to be related to some type of infection.

**IMPORTANCE:**

*Staphylococcus lugdunensis* is a coagulase-negative *Staphylococcus* recognized for its virulence in human clinical infections. However, few studies have focused on investigating the phenotypic and genotypic aspects, as well as the population characteristics, of clinical strains involved in severe invasive infections such as endocarditis or skin and soft tissue infections, or of strains associated with so-called cutaneous carriage. Here, we compared different virulence gene variants in terms of populational aspects and also highlighted important phenotypic trait changes according to the clinical presentation and site of isolation of the strains.

## INTRODUCTION

*Staphylococcus lugdunensis* (SLU) is a type of coagulase-negative staphylococci (CoNS) first described by Freney et al. in 1988 (1). Its capacity to colonize the nasal cavity and inguinal and axillary regions makes it an important component of the human skin microbiome, with a prevalence ranging up to 67% in the global population (2). Despite its strong commensality trait, SLU can also be responsible for various infections, such as osteoarticular infections (OAIs), prosthetic joint infections (PJIs), catheter-related infections, infective endocarditis (IE), and skin and soft tissue infections (SSTIs) (3, 4).

The antimicrobial resistance profile of SLU differs from that of other CoNS strains, as the presence of a BlaZ-type penicillinase, conferring resistance to penicillin G, is found in 25% to 44.6% of strains (5), which is considerably lower than that reported for other CoNS strains (5). In recent decades, resistance to methicillin has become an important clinical issue in CoNS, with the prevalence of *mecA* gene acquisition exceeding 80% in most species (6, 7). Once again, SLU differs from other CoNS, with the prevalence of the *mecA* gene estimated at 1.8% (8). However, several countries in South Asia have recently highlighted the emergence of hospital outbreaks involving strains belonging to clonal complex 1 (CC1), with an estimated methicillin resistance rate of up to 30% (9). The emergence and spread of such epidemic strains could therefore complicate the therapeutic management of infection.

To better understand its particular virulence, the complete genome of SLU has been sequenced, allowing the identification of several virulence and adaptation factors (10). Some of these virulence factors have been characterized using *in vitro* assays, allowing the identification of their roles in bacterial adaptation, colonization, biofilm formation, and bacterial adherence. Among them, several proteins seem to play major roles in human pathogenicity. AtIL is an autolysin involved in cell separation, cell wall remodeling, and cytoplasmic protein excretion (11). Hussain et al. (12) demonstrated its role in biofilm formation. The iron acquisition system is strongly mediated by the SLU iron-regulated surface determinant (Isd), which is composed of seven proteins; one of these proteins, IsdC, also potentially promotes biofilm formation in an iron deficiency model (13). Bacterial adhesion to fibrinogen and the endovascular system involves fibrinogen-binding protein (Fbl) (14), von Willebrand factor-binding protein (vWbl), and sortase A (SrtA) (15). SLU also expresses an accessory gene regulator (Agr) system, enabling virulence regulation according to environmental conditions (16, 17).

Recently, several works have identified the capacity of SLU to synthesize a new bacteriocin called lugdunin, which belongs to the fibupeptide class (18). This antimicrobial peptide can inhibit the growth of other skin commensals such as *Staphylococcus aureus* and could represent an important factor promoting SLU implantation and colonization (18).

Despite the widespread use of next-generation sequencing and easy access to genomic data such as multilocus sequence typing (MLST), virulome, or resistome data, the distribution of these virulence factors throughout SLU populations remains unknown. To our knowledge, limited epidemiological and microbial studies among *S. lugdunensis* populations have led to a better understanding of colonization, antibiotic resistance, virulence, and microbiome interactions. The aim of this study was to identify the genotypic and phenotypic traits of 76 clinical strains of SLU isolated in nine French hospitals associated with carriage or severe infections, including OAIs, IE, or SSTIs. Putative virulence factors and population characteristics were therefore compared in terms of antimicrobial resistance, von Willebrand binding ability, antimicrobial inhibition, biofilm formation assays, and *in vivo* virulence.

## MATERIALS AND METHODS

### Bacterial strains

A panel of 76 nonduplicate *S. lugdunensis* clinical strains from 76 patients was retrospectively collected over a period ranging from January 2001 to December 2016 at nine French hospitals: APHP (Avicennes, Bichat, Necker, and Pitie-Salpetriere), CHU Caen, CHU Lille, CHU Nantes, CHU Rouen, and CH Versailles. Species identification was performed using matrix-assisted laser desorption/ionization-time-of-flight mass spectrometry (Bruker, Germany). The strains were centralized at the microbiology laboratory of Caen University Hospital and stored at −80°C. Epidemiological and clinical data were reported according to age, sex, date of sampling, and origin of infection. Clinical data were available for 70 patients.

### Antimicrobial susceptibility testing (AST)

AST was performed using the disk diffusion method and interpreted in accordance with CA-SFM/EUCAST 2022 recommendations (19). Antibiotic resistance data were obtained for penicillin G, oxacillin, cefoxitin, fosfomycin, erythromycin, fusidic acid, rifampicin, clindamycin, ofloxacin, ciprofloxacin, moxifloxacin, linezolid, vancomycin, kanamycin, and gentamicin.

### DNA sequencing and bioinformatics

Whole-genome sequencing was performed on all *S. lugdunensis* strains using the P2M platform of the Pasteur International Bioresources Network (Institut Pasteur, Paris, France). DNA extraction was performed using a NucleoSpin Microbial DNA Kit (Macherey-Nagel, Germany). Libraries were prepared using a Nextera XT Kit (Illumina, USA), and sequencing was performed on a NextSeq 500 platform (Illumina, USA), generating 150 bp paired-end reads. Alien Trimmer was used for read trimming and clipping (20). The quality of the filtered Fastq files was assessed using FastQC software (https://www.bioinformatics.babraham.ac.uk/projects/fastqc/). Finally, the genomes were assembled *de novo* using SPAdes v.3.12 software with the recommended k-mer sizes for Illumina 150 bp paired reads (-k 21, 33, 55, and 77) (21). The overall quality of the genome assembly was assessed using Quast (22). The species of the sequenced bacteria were verified using the NCBI prokaryotic genome annotation pipeline (23). The sequence type (ST) was determined using MLST on the basis of seven housekeeping genes: *aroE* (shikimate dehydrogenase), *dat* (D-amino acid aminotransferase), *ddl* (D-alanine: D-alanine ligase), *gmk* (guanylate kinase), *ldh* (L-lactate dehydrogenase), *recA* (recombinase), and *yqiL* (acetyl-coenzyme A acetyltransferase) (24). Acquired antibiotic resistance genes and point mutations were identified through the Center for Genomic Epidemiology using the ResFinder and PointFinder databases, respectively (25).

### von Willebrand factor binding assay

An overnight culture of *S. lugdunensis* was diluted in a 0.9% saline solution to obtain a 2 McF concentration. Sterile polystyrene microplates were loaded with 200 µL of bacterial suspension. After centrifugation, the wells were drained to obtain dry bacterial pellets. The wells were then filled with a 0.1% milk solution diluted in phosphate-buffered saline (Thermo Fisher, USA) with 0.05% polysorbate 20. After a 2 h incubation at 35°C ± 2°C, the microplates were centrifuged and drained. The plates were then filled with 200 µL of a 60 UI/mL plasma derived-VWF solution diluted in 0.1% milk. After a 1 h incubation, the microplates were centrifuged and drained. Three flushes were performed before adding 200 µL of 1/8,000 a rabbit polyclonal anti-human VWF antibody coupled with HRP (Dako, Agilent, USA) to the wells. The plates were then incubated for 1 h at 37°C before another centrifugation and three flushes. VWF fixation was revealed with the addition of 200 µL of 3,3′,5,5′-tetramethylbenzidine for 3 min, and the reaction was stopped with sulfuric acid. The absorbance was read at 600 nm. Three independent experiments (each in duplicate) were performed.

### Study of biofilm formation by a crystal violet assay

Overnight cultures of *S. lugdunensis* were diluted in TS broth to an optical density (OD) of 0.1. Sterile polystyrene microplates were then loaded with 100 μL of bacterial suspension and incubated for 24 h at 37°C. Biofilm formation was detected using the method described by Christensen et al. (26). The adherent cells were stained with 0.1% crystal violet for 15 min, and after three washes, the wells were air-dried. For quantitative estimation of biofilm density, bound crystal violet was solubilized with 70% ethanol, and the absorbance of the solubilized dye was read at 600 nm with Tecan Infinite M200 Pro. Three independent experiments (each in duplicate) were performed.

### Phenotypic assay for the determination of antimicrobial inhibition

To determine the potential inhibitory impact of antimicrobial inhibition on several bacterial species, we inoculated agar plates with a 0.5 McF solution of the bacteria of interest. A 10 µL mixture of each *S. lugdunensis* strain with a 1 McF solution was then spotted on agar media. The agar plates were then incubated for 48 h at 35°C ± 2°C in a 5% CO_2_ atmosphere. The inhibition diameter was then measured for each strain. All 76 strains of *S. lugdunensis* were tested against *Micrococcus luteus* ATCC 9341, *Staphylococcus epidermidis* ATCC 10502, *Staphylococcus saprophyticus* ATCC 10507, *Staphylococcus haemolyticus* ATCC 10513, *Staphylococcus hominis* ATCC 10514, *Staphylococcus capitis* ATCC 10508, *Staphylococcus aureus* ATCC 23235, *Staphylococcus aureus* USA 300, *Streptococcus pneumoniae* ATCC 49619*, Enterococcus faecalis* ATCC 29212, *Enterococcus faecium* NCTC 7171, *Escherichia coli* ATCC 25922, *Pseudomonas aeruginosa* ATCC 27853, *Propionibacterium acnes* ATCC 11827, and *Corynebacterium glucuronolyticum* ATCC 51866. Strains were classified as antimicrobial peptide nonproducers if no inhibition was observed, as producers if the size of the inhibition diameter was less than the median of producers, and as high producers if the size of the diameter was greater than the median of producers.

### Virulence study using the *Galleria mellonella* model

Infection of *G. mellonella* larvae by *S. lugdunensis* was performed as described previously by Lebeurre et al. (27). Larvae were infected with a 10 μL injection of *S. lugdunensis* cell suspension (approximately 1 × 10^7^ CFU) obtained from an overnight culture in brain heart infusion broth. Prior to injection, the bacterial pellets were washed and adjusted to the same concentration in saline buffer, and CFUs were counted on agar plates after serial dilutions. For each test, 10 larvae were infected, and the experiments were repeated in triplicate. As a control, sterile saline solution was injected into the larvae. Larval mortality was monitored at 24, 48, and 72 h post-injection.

### Statistical analysis

Qualitative variables were described using counts and percentages. Quantitative variables were summarized as mean ± standard deviation or median with interquartile range (IQR), depending on their distribution. Mean OD values were compared using analysis of variance, followed by Bonferroni-adjusted *post hoc* pairwise comparisons when overall significance was reached. In the virulence study, survival data were analyzed using Kaplan-Meier survival curves, and differences between groups were assessed with the log-rank test. A *P* value <0.05 was considered statistically significant. All statistical analyses were performed using R software, version 4.0 (R Foundation for Statistical Computing, Vienna, Austria).

## RESULTS

### Patient characteristics

The median age of the patients was 62.5 years (IQR, 37.5–62.5), with a sex ratio (M/F) of 0.52. The strains were involved in SSTIs in 39.5% of cases (30/76), OAIs in 26.3% (20/76), IE in 26.3% (20/76), urinary tract infections in 2.6% (2/76), and bloodstream infections in 1.3% (1/76). Carried strains represented only 3.9% (3/76) of this collection (Table S1).

### Genomic epidemiology of the 76 strains in the cohort

**Fig 1:**
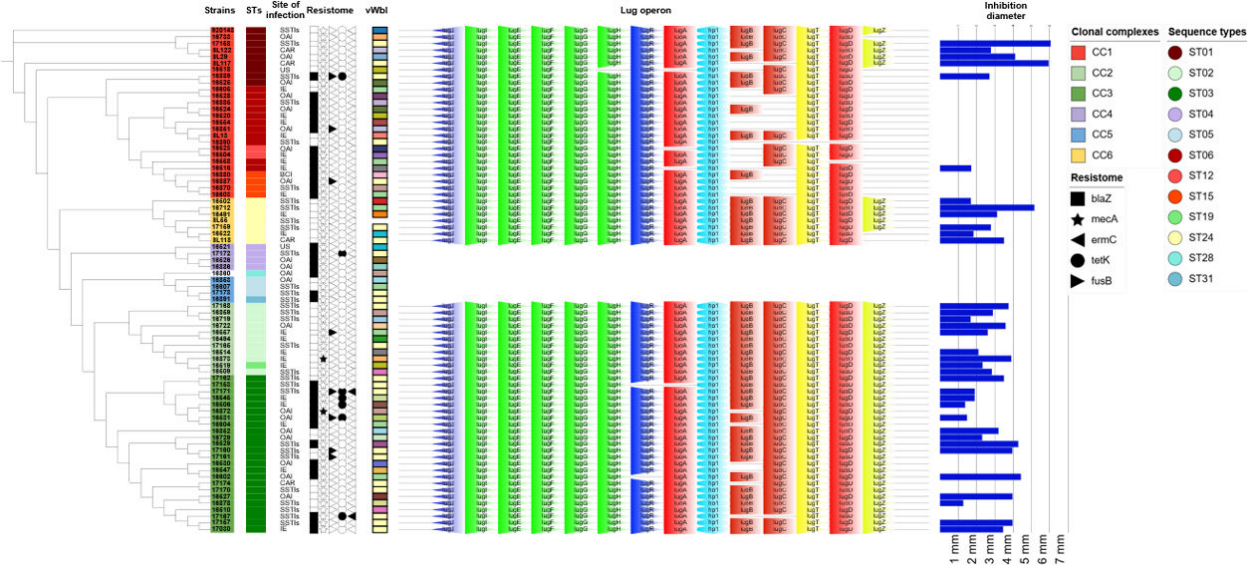
Phylogenetic representation of the *S. lugdunensis* 76-isolate cohort. Phylogenetic networks were constructed using Interactive Tree of Life v.6. Strains were colored according to CCs. Strips were colored according to STs.

MLST analysis revealed 12 STs belonging to six clonal complexes (CC1 to CC6). CC1, CC2, and CC3 were predominant, accounting for 73.6% (59/76) of all strains. One ST, ST28, was not associated with any CCs. All of the phylogenetic data are shown in Fig. 1.

### Antimicrobial susceptibility testing

Penicillin G resistance was detected in 45.7% (38/76) of all strains, methicillin resistance in 2.6% (2/76), erythromycin resistance in 10.5% (8/76), tetracycline resistance in 7.9% (6/76), fusidic acid resistance in 2.6% (2/76), rifampicin resistance in 2.6% (2/76), and vancomycin and linezolid resistance in 0% (0/76). All the phenotypic data were consistent with the genomic resistance patterns, since all penicillin G-resistant strains carried the *blaZ* gene and both methicillin-resistant SLU strains harbored the *mecA* gene (Fig. 1; Table S2). The *blaZ* gene was associated mainly with strains belonging to CC1 (15/25), CC3 (14/24), CC4 (4/4), and CC5 (2/4), whereas this gene was consistently absent from CC2 (0/11) and CC6 (0/7). The *ermC*, *tetK*, and *fusB* genes are associated with erythromycin, tetracycline, and fusidic acid resistance, respectively. These genes were equally distributed among the CCs, except for CC6 strains, for which no resistance gene was found (Fig. 1).

### von Willebrand binding assay and genomic analysis

**Fig 2:**
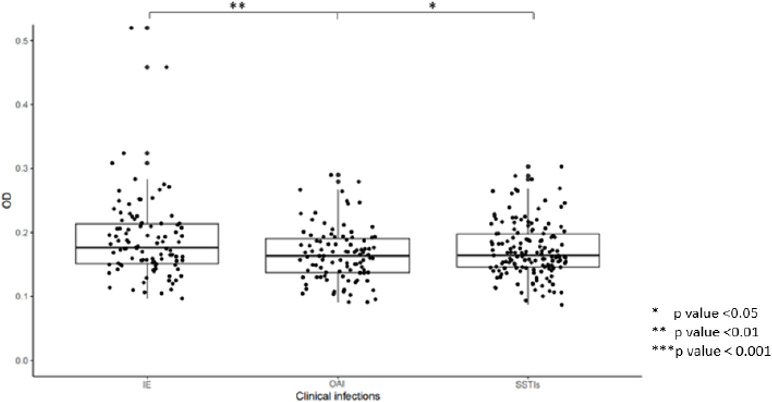
Representation of VWF binding ability according to clinical infection. *P < 0.05, **P < 0.01, and ***P < 0.001.

**Fig 3:**
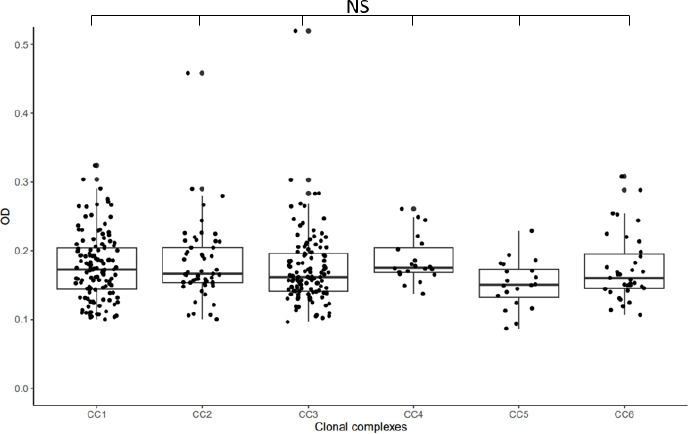
Representation of VWF binding ability according to clonal complexes. NS, not significant.

We deciphered protein variability of all 76 strains by analysis of three genes described as being involved in *Staphylococcus aureus* VWF binding ability: *vWbl*, *srtA*, and *fbl*. First, vWbl was divided into 35 different nucleotide sequences distributed in all CCs without significant difference. Sortase A (encoded by *srtA* gene) was divided into six nucleotide variants associated with CCs. The fibrinogen-binding protein gene (fbl) was divided into 19 nucleotide variants, randomly distributed among CCs. Interestingly, VWF binding ability varied depending on the infectious location of the strains, since strains responsible for IE have a significantly greater capacity of binding than strains isolated from OAI or SSTIs, with OD mean of 0.189 (CI, 0.125–0.253) versus 0.166 (CI, 0.0208–0.124) and 0.172 (CI, 0.13–0.214) (*P* < 0.01) (Fig. 2). No association between von Willebrand binding variants and clinical presentation was found by analyzing these three different virulence factors. No correlation was found between the six CCs and VWF binding ability with OD mean of 0.177 (CI, 0.129–0.224), 0.183 (CI, 0.126–0.240), 0.174 (CI, 0.121–0.227), 0.187 (CI, 0.153–0.221), 0.152 (CI, 0.117–0.187), and 0.175 (CI, 0.126–0.224) (*P* > 0.05) (Fig. 3).

### Biofilm assays and genomic analysis

**Fig 4:**
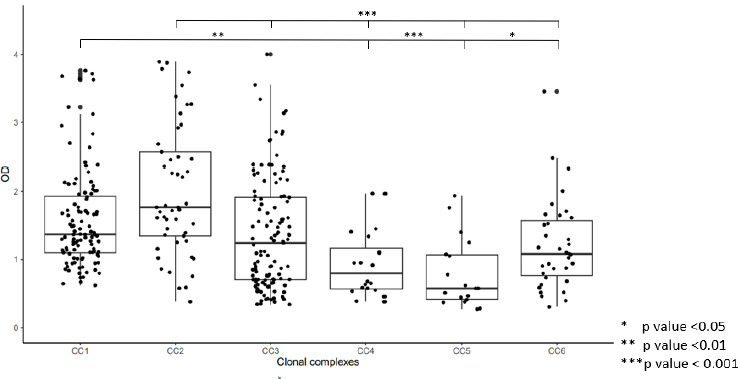
Representation of biofilm production according to different clonal complexes. *P < 0.05, **P < 0.01, and ***P < 0.001.

Interestingly, the study of the 76 strains on a static biofilm formation model using crystal violet staining revealed a significant difference in terms of biofilm production depending on the CCs. A significantly higher biofilm production was observed for CC1 strains compared to CC4, CC5, and CC6 ones (OD mean, 1.27 [CI, 0.95–1.98] vs 0.78 [CI, 0.57–1.17], 0.62 [CI, 0.46–1.08], *P* < 0.01 and 1.07 [CI, 0.78–1.55], *P* = 0.02). In addition, strains belonging to CC2 produced significantly more biofilm (*P* < 0.01) than those of CC3, CC4, CC5, and CC6 (OD mean 1.78 [CI, 1.35–2.95] over 1.27 [CI, 0.95–1.98] vs 0.78 [CI, 0.57–1.17], 0.62 [CI, 0.46–1.08], and 1.07 [CI, 0.78–1.55], *P* < 0.01) as presented in Fig. 4.

**Fig 5:**
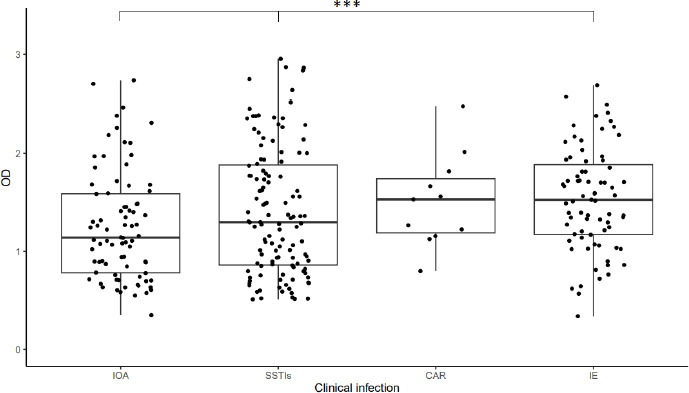
Representation of biofilm production according to different clinical infections. ***P < 0.001.

IE were significantly associated with higher biofilm formation (OD mean, 1.53 [CI, 1.17–1.89]) compared to strains involved in OAI (OD mean, 1.14 [CI, 0.78–1.59] *P* < 0.01) or SSTIs (OD mean, 1.30 [CI, 0.86–1.88]). No difference was highlighted between other infective locations (Fig. 5).

Genomic analyses identified several allelic profiles within the virulence factors known to be involved in biofilm formation such as *agr*, *atIL*, and *isd genes*.

The Agr system, which is known to be involved in quorum sensing, biofilm formation, and overall virulence traits, is encoded by a genomic operon composed of *agrA*, *agrB*, *agrC*, and *agrD* genes. In our panel of strains, a strong nucleotidic sequence homology was found for the *agrA* (only one genetic variant), *agrB,* and *agrD* genes (three genetic variants each). Nevertheless, *agrC* gene sequence was highly variable between the different strains, with more than 13 distinct genetic variants (Fig. S1). Interestingly, each CC has its own membrane effector *agrC* sequence. Eight strains presented a lack of AgrC (two belonging to CC1, two from CC2, one from CC3, two from CC5, and one from CC6) (Fig. S1; Table S4).

Concerning *atIL* gene, a total of 15 variants was found in our panel. Absence of an effective AtIL protein was found in only three strains (e.g., 16870, 16880, and 16887). Sequence distribution was, once again, associated with the different CCs as shown in Fig. S1; Table S4.

In our cohort, the IsdEF transporter and the IsdG heme oxygenase appear to be highly conserved. Conversely, IsdB, IsdJ, and IsdK showed a different allelic distribution between CCs (Fig. S1; Table S5).

### Antimicrobial ability

A study of the inhibition ability of *S. lugdunensis* strains on solid medium revealed an inhibition diameter for 49% (37/76) of the strains against *Micrococcus luteus* (Picture S1). Interestingly, these 37 *S. lugdunensis* strains had an inhibitory effect against the reference CoNS strains tested, such as *S. haemolyticus*, *S. capitis*, *S. epidermidis*, and *S. saprophyticus*. Nevertheless, no inhibition was observed against the *S. aureus*, *E. faecalis*, *E. faecium*, *S. pneumoniae*, *E. coli*, *Enterobacter cloacae*, *P. aeruginosa, C. glucuronolyticum*, or *P. acnes* strains. Therefore, genomic analyses revealed an absence of the lug operon and no inhibition diameters for strains from CC4 and CC5. Among the four main CCs, there was a particular distribution of inhibition ability against all CoNS since consistent inhibition diameters were found for 32% (8/25), 100% (10/10), 58% (14/24), and 100% (7/7) of the strains belonging to CC1, CC2, CC3, and CC6, respectively. The distribution of the ability of these inhibitors was homogeneous in each type of infection, although constant inhibition ability was found in carriage strains (3/3) (Fig. 1; Fig. S2; Tables S3 and S6).

### *In vivo* virulence of the SLU strains

**Fig 6:**
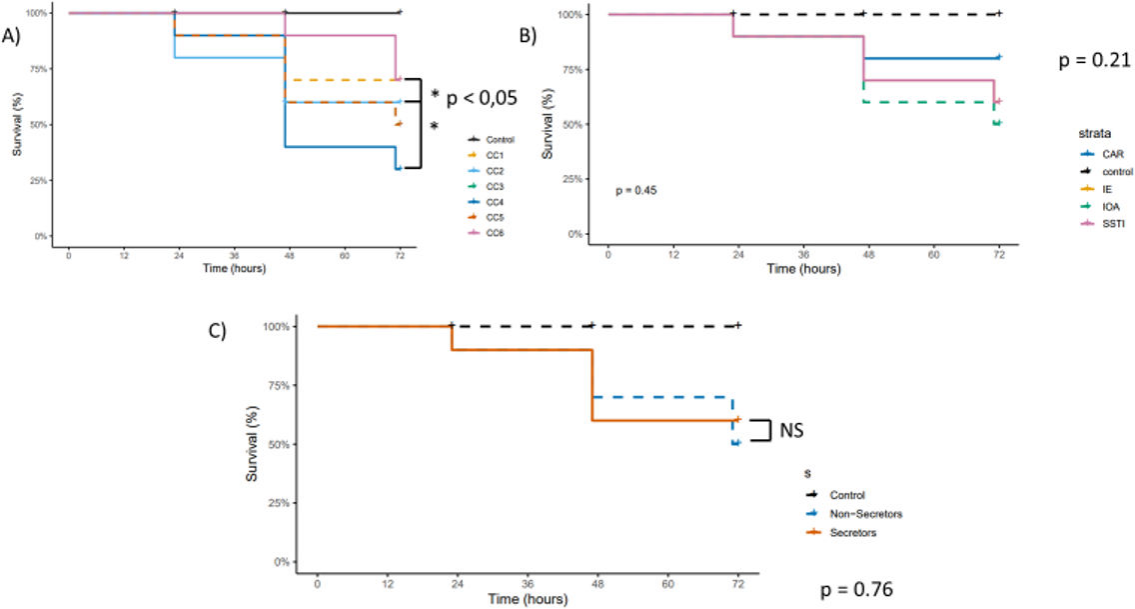
Mortality curves according to the different CCs (**A**), clinical locations (**B**), and lugdunin secretors (**C**). CAR, healthy carriage strains; IE, strains responsible for infective endocarditis; IOA, strains responsible for osteoarticular infection; SSTI, strains responsible for skin and soft tissue infections. *P < 0.05; and NS, not significant.

In the *G. mellonella* infection model, larval mortality was similar for strains belonging to CC1, CC2, CC3, CC5, and CC6. Nevertheless, strains belonging to CC4 caused significantly greater mortality than strains belonging to other CCs, such as CC1, CC2, and CC6 (*P* < 0.05) (Fig. 6A). Analysis of mortality according to clinical location did not reveal any significant difference. Mortality in the “carriage” group tended to be lower (*P* = 0.21) than that in the invasive infection group; however, the result was not statistically significant (Fig. 6B). The mortality of lugdunin-producing strains versus nonproducing strains did not significantly differ (*P* = 0.71) (Fig. 6C).

## DISCUSSION

*S. lugdunensis* is a staphylococcal species that harbors a distinct resistome and original pathogenic traits among coagulase-negative staphylococci, likely explained by singular virulence factors.

In our cohort, the 76 strains were distributed across six CCs (CC1 to CC6) and one singleton (ST28), suggesting that our strains appear to be representative of the overall SLU population described to date (24). Indeed, subsequent studies have identified seven clonal complexes, with CC1 to CC3 appearing to be predominant (24, 28). Analysis of the different STs leading up to these CCs supported the idea that there was no association between clinical infection types and phylogenetic lineages (28).

The role of vWbl in *S. aureus* adhesion to von Willebrand factor during endothelial damage has been linked to endocardial pathogenicity (29). Liesenborghs et al. demonstrated that SLU adheres to VWF more effectively than other coagulase-negative staphylococci in both microvascular perfusion and murine endocarditis models (30). The present study confirmed enhanced VWF binding among SLU strains involved in infective endocarditis. Analysis of vWbl, sortase A, and Fbl sequences revealed clonal distribution of mutations, as previously described, with protein conservation within each clonal complex (28, 31). However, VWF binding capacity was present across all strains regardless of clonal complex or variations in sortase A, vWbl, or Fbl, suggesting that additional virulence factors contribute to VWF adhesion (30).

Here, we demonstrate the capacity for biofilm formation of strains belonging to CC1 and CC2. This ability to form biofilms led us to hypothesize that these CCs are able to establish themselves predominantly in their environment because of this competitive advantage. This competitive advantage is reflected in the presence of virulence factors specific to each complex. However, the large number of these factors involved in the biofilm (10) did not establish an association between gene content and biofilm formation.

The *G. mellonella* infection model revealed higher mortality induced by strains belonging to CC4. Interestingly, all the strains from CC4 isolated in our cohort were responsible for infections, reinforcing the hypothesis of a singular virulence of this clonal complex. The fact that CC4 was rare in the infectious environment (28) could be due to a lack of lugdunin production, and we hypothesize that its particular virulence is essential for strain persistence. Several studies have shown the role of lugdunin in conferring a competitive advantage *to S. lugdunensis* over *S. aureus* in the context of skin and nasal colonization (18). This ability is predicated on the direct bactericidal effect of lugdunin against *S. aureus* and an additional mechanism involving immune conditioning of epithelial cells by lugdunin (32).

We showed that SLU inhibition ability was extremely variable between the different CCs due to the loss of all or some of the *lug* operon genes. Interestingly, strains belonging to CC1, CC2, and CC3 were predominant among the inhibitor strains (83.7%). As these clonal complexes are also involved in most infections, it can be hypothesized that the production of lugdunin provides a significant competitive advantage to CC1, CC2, and CC3, thus enabling their predominance over other CCs (Fig. S2). However, strains with inhibition ability possess virulence traits analogous to those of others, which lends further credence to the hypothesis that this peptide plays a pivotal role exclusively in a colonization mechanism. Genomic and *in silico* analyses revealed significant genetic features in the lugdunin production machinery (Fig. 1).

Comparative analysis of production profiles and allelic diversity revealed the functional organization of the lugdunin biosynthetic operon. The nonribosomal peptide synthetases encoded by *lugABCD* constitute four essential protein modules required for lugdunin biosynthesis; loss of any single module abolishes production. The *lugR* regulator, located upstream of the *lugABCD* operon, was mutated in two CC3 strains (16602 and 17153). Notably, loss of *lugR* in the presence of functional biosynthetic machinery consistently increased the zone of inhibition, suggesting *lugR* functions as a negative regulator. The remainder of the operon comprises *lugIEFGH,* encoding ABC transporters involved in lugdunin secretion and self-resistance; *lugJ*, encoding a transcriptional regulator; and *lugT*, *lugZ*, and *lugM*, encoding modification enzymes (4, 18).

Systematic screening of genes encoding putative virulence factors revealed associations between allelic variation and clonal complexes. The Agr quorum-sensing system is directly involved in *S. aureus* pathogenicity, where high population variability in *agr* influences infection type (33). This variability predominantly affects the AgrB transpeptidase, AgrC membrane receptor, and AgrD autoinducing peptide (34). In the present cohort, variability was predominant in AgrB and AgrC proteins. The two-component response regulator AgrA was highly conserved, suggesting it is essential for *S. lugdunensis* virulence expression. However, no correlation between Agr allelic profile and clinical infection type was established in this cohort.

The Isd system is composed of seven proteins involved in iron uptake and biofilm formation. The protein structure showed predominant variability in IsdB, IsdJ, and IsdK, suggesting the relative importance of heme binding to these membrane proteins does not appear to have a fundamental role in iron acquisition given their high variability. On the other hand, IsdC appears to be particularly conserved, and its crucial importance in biofilm formation could be important for the species. IsdG is the most highly conserved protein, with only three different allelic variants noted in our cohort. Haley et al. (35) demonstrated the central role of IsdG in heme degradation when IsdG mutants were unable to use heme as an iron source for growth. The importance of the IsdEF transporter required for heme internalization also seems to be an essential part of the system highlighted by its high level of conservation (36).

The AtIL autolysin required for immune escape and biofilm production (12, 37) presented allelic variation that perfectly matched the clonal complexes of SLU. Here, again, the evolution of this autolysin appears to be specific to each CC. Finally, this highly conserved trait also differs between *S. lugdunensis* CCs.

### Limitations

Although based on a multicentric large cohort, the number of strains remains relatively scarce for robust genotype-phenotype associations, especially when subdivided by clonal complex and clinical syndrome. In addition, clinical information at the patient level is only partially integrated, which reduces the ability to fully relate the microbiological findings to clinical outcomes. Nevertheless, the study includes a substantial number of well-characterized strains with comprehensive genomic and phenotypic analyses, providing valuable insights and significantly advancing our understanding of *S. lugdunensis* diversity and virulence.

### Conclusion

Our study, which was based on a large cohort of *S. lugdunensis*, established a correlation between clonal specificities and phenotypic traits. The study of VWF binding ability revealed a correlation between endocarditis and the ability to bind VWF. The phenotypic and genotypic study of the lugdunin operon revealed antimicrobial activity, which varies between different strains, and supported its important role as a competitive advantage of SLU. This colonization also seems to involve other clonally distributed virulence factors associated with biofilm formation. Finally, we demonstrated that CC4 appears to be more virulent.

Further genomics studies of other virulence factors, such as those involved in environmental attachment (e.g., adhesins and MSCRAMs) (38), could provide new data concerning the potential mechanisms of dissemination in the *S. lugdunensis* population.

## Data Availability

*In silico* analyses were performed using publicly accessible and published algorithms/software. The parameters used are detailed in the Materials and Methods. All genomics data are available at BioProject under the identification number PRJNA1182231. All raw data obtained during the different experiments are available following the link https://doi.org/10.6084/m9.figshare.30365350.

## SUPPLEMENTAL MATERIAL

Picture S1 spectrum.03856-25-s0001.tiff *S. lugdunensis* inhibition ability against *M. luteus*.

Figure S1 spectrum.03856-25-s0002.tif Phylogenic representation of the 76 *S. lugdunensis* isolates.

Figure S2 spectrum.03856-25-s0003.tif Population characteristics of lugdunin producers and non-producers.

Supplemental tables spectrum.03856-25-s0004.xlsx Tables S1 to S6.

ASM does not own the copyrights to Supplemental Material that may be linked to, or accessed through, an article. The authors have granted ASM a non-exclusive, world-wide license to publish the Supplemental Material files. Please contact the corresponding author directly for reuse.
